# Remora cranial vein morphology and its functional implications for attachment

**DOI:** 10.1038/s41598-017-06429-z

**Published:** 2017-07-19

**Authors:** Brooke E. Flammang, Christopher P. Kenaley

**Affiliations:** 1 Department of Biological Sciences, New Jersey Institute of Technology, University Heights, Newark, NJ 07102 USA; 2 Department of Biology, Boston College, Chestnut Hill, MA 02467 USA

## Abstract

Remora fishes adhere to, and maintain long-term, reversible attachment with, surfaces of varying roughness and compliance under wetted high-shear conditions using an adhesive disc that evolved from the dorsal fin spines typical of other fishes. Evolution of this complex hierarchical structure required extensive reorganization of the skull and fin spines, but the functional role of the soft tissues of the disc are poorly understood. Here I show that remora cranial veins are highly-modified in comparison to those of other vertebrates; they are transposed anteriorly and enlarged, and lie directly ventral to the disc on the dorsum of the cranium. Ancestrally, these veins lie inside the neurocranium, in the dura ventral to the brain, and return blood from the eyes, nares, and brain to the heart. Repositioning of these vessels to lie in contact with the ventral surface of the disc lamellae implies functional importance associated with the adhesive mechanism. The position of the anterior cardinal sinus suggests that it may aid in pressurization equilibrium during attachment by acting as a hydraulic differential.

## Introduction

Remoras (Echeneoidea) comprise a clade of fishes that have evolved an adhesive disc on the dorsal aspect of the head which permits them long-term reversible attachment to a variety of hosts of different roughness and body compliance (including sharks, rays, whales, dolphins, marlin, turtles, other remoras, divers, and boats)^1–6^.

Whereas previous research has investigated the development of the remora disc^7^, adhesive force measurements^8,9^, and the skeletal components contributing to the hierarchical mechanics of adhesion^8,10^, little attention (with the exception of the anatomical description of the epithelium, muscles, and nerves of the remora disc by Houy^11^) has been paid to the soft tissues that contribute to remora adhesion mechanics^12^.

Here I describe a previously unidentified circulatory structure in the remora adhesive mechanism that may contribute to the versatile attachment abilities of remoras.

## Results and Discussion

**Figure 1:**
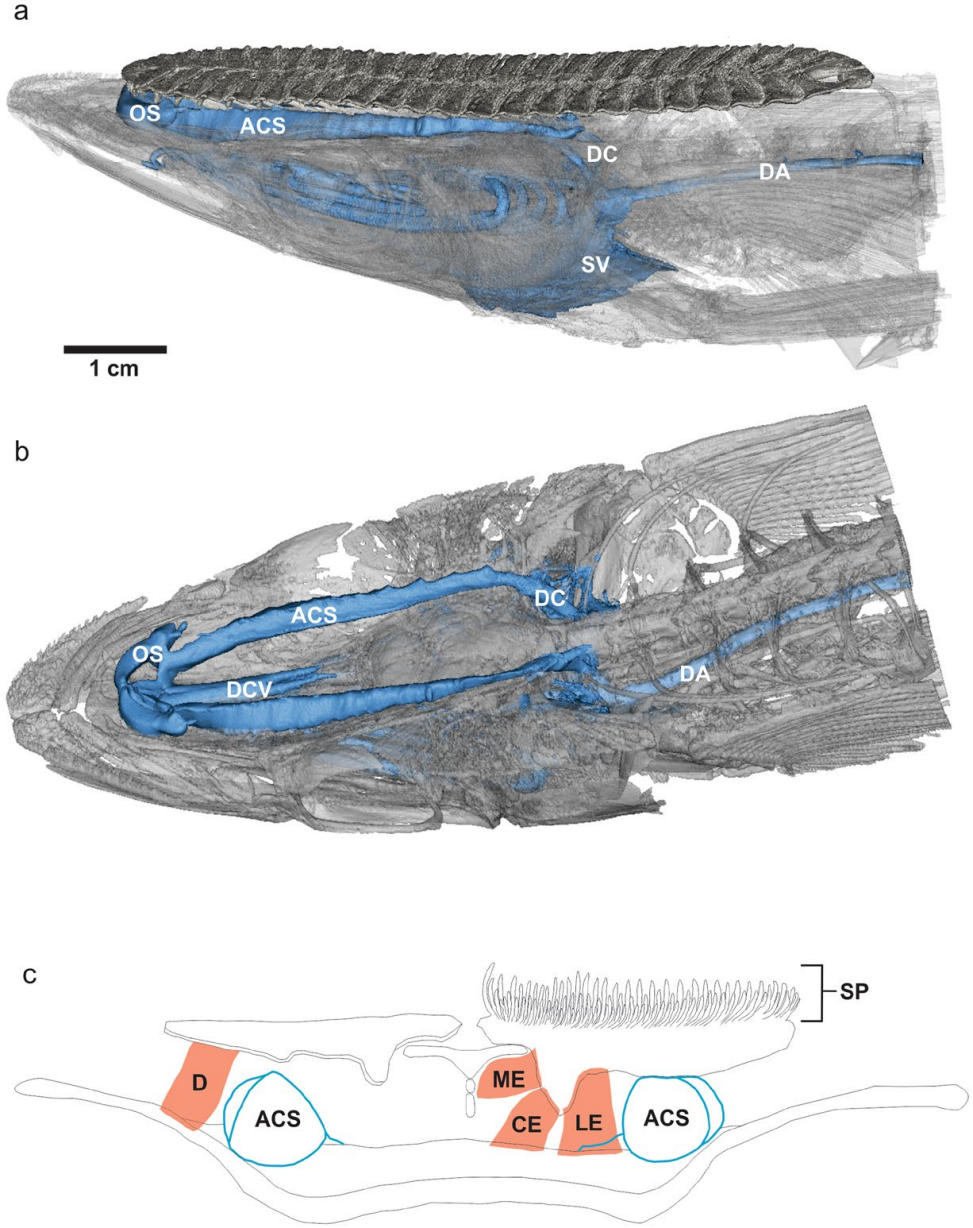
Cranial veins of the remora, *Echeneis naucrates*, post-perfusion with iodixanol (blue). (**a)** Lateral view. (**b)** Dorsolateral view, disc digitally removed. ACS, anterior cardinal sinus; DA, dorsal aorta; DC, ductus Cuvieri; DCV, dorsal collector vein; OS, orbital sinus; SV, sinus venosus. (**c**) Diagram of transverse view or remora cranium at mid-orbit, left lamellae is depressed, right lamellae is erected. CE central erector muscle; D, depressor muscle; LE, lateral erector muscle; ME, medial erector muscle; SP, spinules. In this orientation, spinules are not visible on the depressed lamellae. An interactive model of the micro CT scan is available both as a 3D PDF in Supplementary Materials and online here: https://skfb.ly/6oLuR.

Dissection of a euthanized, fresh remora revealed large diameter blood vessels lying dorsal to the skull and in contact with the ventral side of the lamellae and associated musculature of the adhesive disc. This vasculature was better visualized through reconstruction of computed microtomography (µCT) scans of remora specimens perfused with iodixanol, which revealed that these exceptionally large cranial veins (approximately 2.8–3.3 mm in diameter, or 11.2–13.2% of head width) are located within a space situated between the pectinated lamellae of the disc and the dorsal aspect of the cranium (Fig. 1), indicating that when the lamellae are erected, the cranial veins and their overlying epithelium constitute the roof of each lamellar compartment when attached to a host organism. The large blood vessels are flanked laterally and medially by the erector and depressor muscles that control the rotation of the lamellae.

In the remora (Fig. 1), blood leaves the neurocranium anteriorly through the dorsal collecting vein (DCV) and from the eyes via the orbital sinus (OS), and passes through the anterior cardinal sinus (ACS), through the ductus Cuvieri (DC) to return to the sinus venosus (SV) of the heart. Blood is then pumped through the gills to be oxygenated and returns to the body through the dorsal aorta (DA).

Notably, in other vertebrates^13–15^, the ACS is located primarily inside the neurocranium ventral to the brain in the dura. In humans it is known as the cavernous sinus; the primary function of cavernous sinus is to return blood from the brain, eyes, and nares through the superior and inferior petrosal sinus to the jugular vein (homologous to the ductus Cuvieri^16^), back to the heart. In cobia (*Rachycentron canadum*), the closest living relative of remoras, the ACS is in the dura, ventral to the brain, and measures 0.84 mm in diameter (3.4% of head width) at its largest point as it exits the neurocranium through the jugular foramen.

The dorsally and anteriorly transposed position of the cranial vessels of remoras shows considerable morphological deviation from the normal, protected position of the thin-walled ACS within the neurocranium as compared to all other vertebrates, indicating that this vessel has an important functional role driving its anomalous location. Divergence from the general vertebrate cranial vasculature condition is not entirely unique to remoras; highly-modified cranial vessels form the brain-warming rete in mobulids^17^ and eye-warming rete in lamnid sharks^18^. However, remoras adhere to both exothermic and endothermic hosts, thereby making function as a heat-sink unlikely as a driving factor in this evolutionary novelty. Instead, it is quite likely that the highly-modified remora ACS has a functional role in the cranial adhesive mechanism.

**Figure 2:**
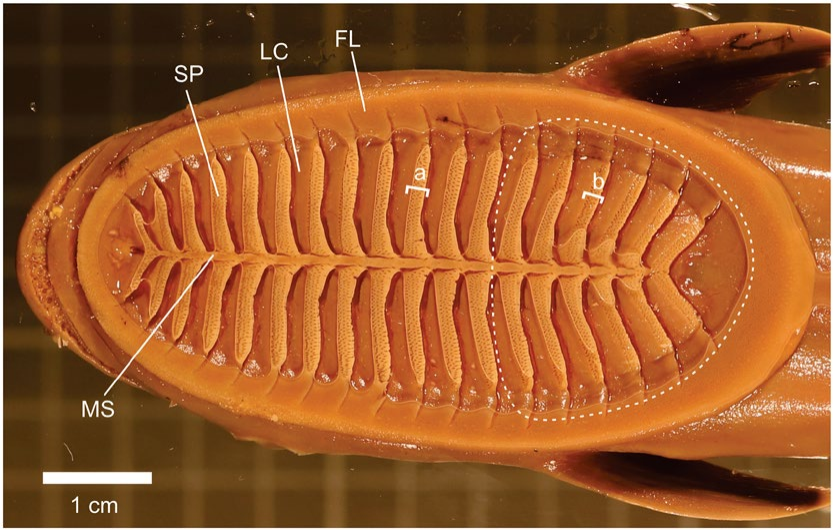
Disc of a remora attached to glass; tissue appears lighter in color when it is sealed to the glass. The fleshy epithelial lip (FL) surrounds the edge of disc and creates the suction seal. The pectinated lamellae (PL) rotate to contact the host, generating friction when spinules interact with local surface asperities. A lamellar compartment (LC) is created between two neighboring rotated lamellae and is sealed on four sides by the thick epithelium on the distal edge of the lamellae (**a**), fleshy lip, and surface of the medial septum (MS). Note that in the more posterior region of the disc (white dashed circle), the lamellae do not create a full seal with the host surface (**b**).

**Figure 3:**
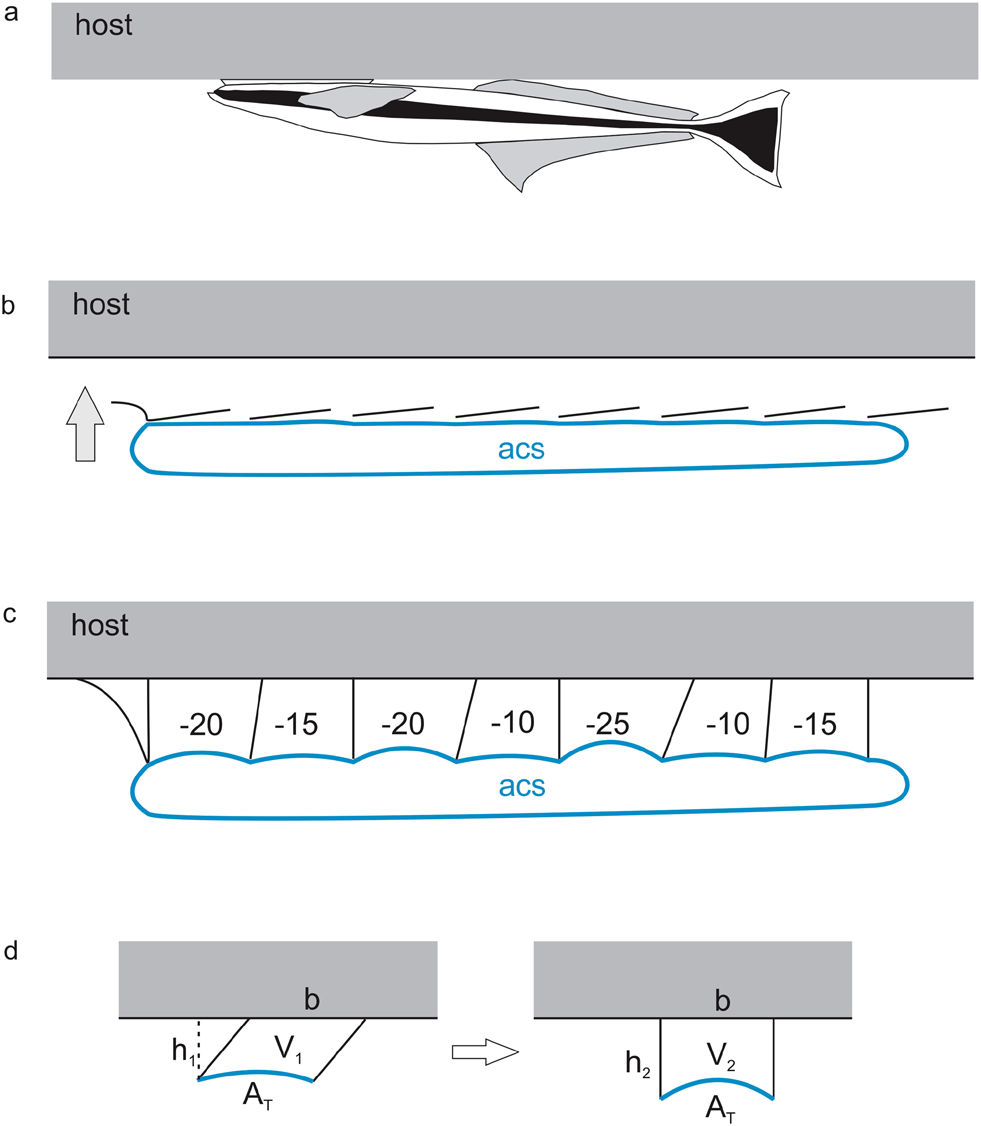
The remora anterior cardinal sinus as a hydrostatic differential. (**a**) Remora attached to a host. (**b**) Remora approaching a host, lamellae depressed before contact is made. (**c**) Host organisms all have some degree of surface roughness or curvature, therefore, in attachment to a host, not all lamellae rotate to the same degree, thus not all lamellar compartments are equal in volume or pressure. The thin-walled anterior cardinal sinus (ACS) is deformed by lamellar rotation and thereby could equalize pressure differences among lamellar compartments within the disc. Numerical values illustrate hypothesized variation in pressure differentials within the disc. (**d**) Bulging of the ACS into the lamellar compartment (A_T_) occurs passively during lamellar rotation when compartment height (h) increases, causing compartmental volume to increase (V), whereas the host surface area of any given lamellar compartment (**b**) remains constant.

Remora adhesion is primarily achieved by friction and suction. Frictional force is generated by the small spinules of the pectinated lamellae (SP, Fig. 2) that interact with host surface to overcome, by an order of magnitude^8^, the drag experienced by remoras. Therefore, the most probable cause of failure over long periods of hold time is not slippage but loss of suction as a result of seep^12^. Suction under the disc is achieved by rotation of the pectinated lamellae when the disc is in contact with the host – this creates a relatively negative, sub-ambient pressure space under the disc^8,9^. The edge of the disc is sealed by a fleshy lip of thick epithelium, as are the edges of the lamellae and the median septum, thereby creating a sealed compartment between two neighboring lamellae (Fig. 2a). Suction failure results when fluid seeping under the fleshy edge of the disc causes equalization between under-disc and ambient pressures^19^. Host organisms to which remoras adhere have curved bodies with varying ranges of surface roughness, resulting in uneven attachment surfaces. Lamellar rotation is individually controlled to permit the disc to shape its contact surface to the host, which results in unequal volume compartmentalization between lamellae such that the initial pressure within a lamellar compartment would not be equal to that of its neighbors (Fig. 3a). Unequal pressurization in neighboring lamellar compartments would promote seep from outside the disc leading to equilibrium^12^; thereby causing loss of suction adhesion.

I hypothesize that the antero-dorsally transposed position of the ACS allows it to function as a hydraulic differential^20^, promoting equalization of pressure among lamellar compartments at the time of attachment, thereby eliminating localized relative pressure gradients that would increase fluid seep and cause suction failure. Blood vessels have non-linearly elastic properties and exhibit strain-dependent increases in elasticity and stiffness^21–23^. The ACS is a thin-walled vein unlikely to resist deformation as a result of pressurization. Functioning as a hydraulic differential, the dorsal wall of the ACS would bulge into the lamellar compartment in proportion to the sub-ambient pressure within any given compartment (Fig. 3), thereby rendering the average pressure throughout the space between the disc and the host relatively uniform. Eliminating the pressure differential among lamellar compartments resists seepage by removing focal weak points along the disc edge. This mechanism may be especially important when the host surface is irregular or curved, as it is on host organisms to which remoras adhere, in which case, variation in volume among lamellar compartments would be greater. Incidentally, the hierarchical adhesive mechanism of some geckos also relies upon a fluid pressure differential; the reticular network of enlarged blood vessels in the lamellar plates allows deformation of scansors along multiple axes at a single time, thereby promoting adhesive contact on irregular surfaces^20,24,25^.

It is worth noting, however, that the ACS only lies deep to the anterior 70% of the disc; the more caudal 30% of the disc lies dorsal to the body. If this posterior 30% of the disc was also functionally divided into separate lamellar compartments, it is reasonable to expect that this portion of the disc would be more susceptible to seep because it lacks the hydraulic differential system for equilibration. However, during adhesion, the posterior region of disc is not divided into discrete lamellar compartments, because those lamellae do not create a seal with the host surface (Fig. 2,b). By injecting food dye under the disc of a live, attached remora, I found that fluid is contained within any of the individual lamellar compartments in the anterior 70% of the disc, but dye injected between lamellae in the posterior 30% of the disc circulates within this posterior region. This suggests functional regionalization within the disc; the posterior-most lamellae are not as tightly associated with the host and the posterior region acts as one continuous suction component. The large, posterior suction region of the remora disc is advantageous in two ways: the fleshy lip is wider and it has a larger cavity volume. The fleshy lip of the disc is wider in the posterior region than the anterior region (Fig. 2) and, following Darcy’s law^26^, this requires a larger pressure gradient to induce seep across a longer seal distance. And, because the larger posterior cavity takes longer to fill with a given seep rate, it extends the time to attachment failure.

The remora adhesive disc is a functional novelty that is the product of extensive modulation of pre-existing morphology in its evolution. In cobia, the closest living relative of remoras^27^, the dorsal fin spines are located approximately 40–45% of total length, measured from the rostrum; the remora adhesive disc evolved from fin spines like those in cobia. The most basal remora (^†^*Opisthomyzon*) possessed a disc-like structure with fewer lamellae (which lacked spinules) and was located farther caudally than modern remoras^27–29^. This suggests that basal remoras may have had adhesive abilities, albeit potentially limited, before the disc was transposed anteriorly and superficial to the cranium. Therefore, reorganization of the cranial vasculature was independent of the exaptation of the dorsal fin elements in forming the adhesive disc. Development of the remora adhesive disc does not appear to modify the development of the neurocranium^7^, suggesting that modulation of the cranial veins was independently derived. The evolution of this unique morphology, that would otherwise seem constrained given the nature of its physiology, and which places a large, thin-walled vein in a very superficial position anatomically, underlies the functional importance of the ACS in the remora adhesive mechanism.

## Experimental Procedures

Remoras (*Echeneis naucrates*) were kept in 1135 L aquaculture tanks with 12:12 hour light:dark cycle in the aquatic animal facility in the Department of Biological Sciences at the New Jersey Institute of Technology, and in the Lauder Laboratory at Harvard University during pilot studies. They were fed a custom fish, shrimp meal, and vegetable mixture daily. All animals were handled ethically according to institutional animal care and use protocols approved by the New Jersey Institute of Technology (14–036) and Harvard University (20–03).

One remora was euthanized with an overdose of tricaine methanesulfonate (Tricaine-S, Western Chemical, Inc., Ferndale, WA) and dissected in preparation for an electromyography study, which led to my initial discovery of the novel vein structure described here.

Two remora (24 and 33 cm TL) were used for imaging cranial vasculature. Transcardiac perfusion was performed after fish had been euthanized by an overdose of Tricaine-S. An incision was made between the dentaries and pectoral girdle and the skin reflected to reveal the heart. A 26 g needle attached to polyethylene outlet tubing of a small peristaltic pump (P720, Instech Laboratories, Plymouth Meeting, PA) was inserted into the conus arteriosus and 30–50 ml of heparinized saline (1 mg/ml)^30^ perfused, depending upon the size of the fish. A small cut was made in the sinus venosus to drain blood and fluids so as not to overinflate vessels and risk bursting. For contrast, 50–70 ml of iodixanol (270 mg/ml, Visipaque 270, GE Healthcare Inc., USA) was then perfused and either µCT scanned immediately or placed in formalin and µCT scanned within 18 hours (dependent upon scanner availability) in a Skyscan 1173 (125 kV, 48 µA, 35.17 µm pixel size, MicroPhotonics, Inc., USA) or Skyscan 1275 (80 kV, 125 µA, 31.00 µm pixel size, MicroPhotonics, Inc., USA). Reconstruction and segmentation of µCT scans was performed in Mimics 18 (Materialise USA).

One remora (37 cm TL) was lightly anaesthetized using Tricaine-S to permit handling without inducing stress, and placed onto a piece of acrylic into which a 1.5 mm hole had been drilled and polyethylene tubing had been glued using cyanoacrylate. The remora was positioned so that a single lamellar chamber was lying over the hole and blue food dye was injected into the lamellar compartment. The dye was observed for 1 minute and then the remora was moved off the plate to clear the dye. The procedure was replicated for each lamellar chamber in the disc. The remora was under light sedation for 28 minutes, was observed in a revival tank for one hour, and returned to its tank without incident.

Two cobia (*Rachycentron canadum*), one preserved (30 cm TL) and one received fresh frozen (28 cm TL; Rosenstiel School of Marine and Atmospheric Science, University of Miami), were dissected to measure the ACS for comparison to the remora. In both cases, a midsagittal incision was made in the cranium using a Dremel rotary tool and the ACS located at the jugular foramen in the posterior neurocranium.

### Data availability

The datasets generated during and/or analysed during the current study are available from the corresponding author on reasonable request.

## Electronic supplementary material

Supplementary Information
