# Author Correction: Remora cranial vein morphology and its functional implications for attachment

**DOI:** 10.1038/s41598-018-20761-y

**Published:** 2018-02-12

**Authors:** Brooke E. Flammang, Christopher P. Kenaley

**Affiliations:** 1 Department of Biological Sciences, New Jersey Institute of Technology, University Heights, Newark, NJ 07102 USA; 2 Department of Biology, Boston College, Chestnut Hill, MA 02467 United States

Correction to: *Scientific Reports* 10.1038/s41598-017-06429-z, published online 19 July 2017

Christopher P. Kenaley was omitted from the author list in the original version of this Article. This has been corrected in the PDF and HTML versions of the Article.

The Acknowledgements section now reads:

BEF wishes to thank E.J. Anderson, J.H. Nadler, M. Beckert, A.P. Summers, and G.V. Lauder for their encouraging discussions. A.P. Russell offered tremendous insight that greatly improved the quality of this manuscript. K. Hartel and A. Williston (Museum of Comparative Zoology, Harvard University) generously loaned specimens for comparative observation. S. Crofts, C. Crawford, K. Gamel, L. Winn, and A. Tam assisted in the dye injection experiments. K. Suri and K. Stanchak assisted in several perfusion experiments and CT scans. D. Benetti and J. Fiorentino (RSMAS, University of Miami) provided the cobia. B. Ache and B. Walters (MicroPhotonics, Inc.) assisted in μCT scanning.

The Author Contributions section now reads:

BEF discovered the anatomy, conceived and conducted the experiments, analysed the data, developed the functional theory, prepared the figures, and wrote the manuscript. CPK provided the contrast agent and operated the CT scanner for the experiment that resulted in Figure 1.

