# Supplementary material for: Remora cranial vein morphology and its functional implications for attachment
Source: Sci Rep. 2017 Jul 19;7:5914. doi: 10.1038/s41598-017-06429-z (PMC5517627; doi:10.1038/s41598-017-06429-z)
Supplement: Supplementary file 1 — Supplementary Information [file 41598_2017_6429_MOESM1_ESM.pdf]

# Remora cranial vein morphology and its functional implications for attachment

[1]Brooke E. Flammang and [2]Christopher P. Kenaley

[1]Department of Biological Sciences, New Jersey Institute of Technology, University Heights, Newark, NJ USA 07102

[2]Department of Biology, Boston College, Chestnut Hill, MA 02467

Author for correspondence: Brooke Flammang (flammang@njit.edu)

**Supplementary Data 1.** Three-dimensional reconstruction of microCT scan of a remora (*Echeneis naucrates*) cranium following perfusion with iodixanol; orange, adhesive disc; blue, cranial veins; grey, skull.
